# FAM20: an evolutionarily conserved family of secreted proteins expressed in hematopoietic cells

**DOI:** 10.1186/1471-2164-6-11

**Published:** 2005-01-27

**Authors:** Demet Nalbant, Hyewon Youn, S Isil Nalbant, Savitha Sharma, Everardo Cobos, Elmus G Beale, Yang Du, Simon C Williams

**Affiliations:** 1 Department of Cell Biology and Biochemistry, Texas Tech University Health Sciences Center, Lubbock, Texas 79430, USA; 2 Department of Internal Medicine, Texas Tech University Health Sciences Center, Lubbock, Texas 79430, USA; 3 Southwest Cancer Center at University Medical Center, Lubbock, Texas 79430, USA

## Abstract

**Background:**

Hematopoiesis is a complex developmental process controlled by a large number of factors that regulate stem cell renewal, lineage commitment and differentiation. Secreted proteins, including the hematopoietic growth factors, play critical roles in these processes and have important biological and clinical significance. We have employed representational difference analysis to identify genes that are differentially expressed during experimentally induced myeloid differentiation in the murine EML hematopoietic stem cell line.

**Results:**

One identified clone encoded a previously unidentified protein of 541 amino acids that contains an amino terminal signal sequence but no other characterized domains. This protein is a member of family of related proteins that has been named family with sequence similarity 20 (FAM20) with three members (FAM20A, FAM20B and FAM20C) in mammals. Evolutionary comparisons revealed the existence of a single FAM20 gene in the simple vertebrate *Ciona intestinalis *and the invertebrate worm *Caenorhabditis elegans *and two genes in two insect species, *Drosophila melanogaster *and *Anopheles gambiae*. Six FAM20 family members were identified in the genome of the pufferfish, *Fugu rubripes *and five members in the zebrafish, *Danio rerio*. The mouse Fam20a protein was ectopically expressed in a mammalian cell line and found to be a bona fide secreted protein and efficient secretion was dependent on the integrity of the signal sequence. Expression analysis revealed that the Fam20a gene was indeed differentially expressed during hematopoietic differentiation and that the other two family members (Fam20b and Fam20c) were also expressed during hematcpoiesis but that their mRNA levels did not vary significantly. Likewise FAM20A was expressed in more limited set of human tissues than the other two family members.

**Conclusions:**

The FAM20 family represents a new family of secreted proteins with potential functions in regulating differentiation and function of hematopoietic and other tissues. The Fam20a mRNA was only expressed during early stages of hematopoietic development and may play a role in lineage commitment or proliferation. The expansion in gene number in different species suggests that the family has evolved as a result of several gene duplication events that have occurred in both vertebrates and invertebrates.

## Background

Hematopoietic differentiation is a complex process whereby multiple functionally and morphologically distinct cell types arise from a population of pluripotent hematopoietic stem cells (PHSCs) [1]. The accurate and efficient regulation of hematopoietic development is controlled by a large number of regulatory proteins that have been identified over the past few decades. These regulatory molecules include the hematopoietic growth factors (HGFs), soluble proteins that recognize specific receptors on the surface of sub-populations of hematopoietic cells, thereby initiating signal transduction pathways that modulate the differentiation, proliferation, and/or survival of target cells [2]. The identification of regulators of hematopoiesis has been an ongoing effort for many years and has benefited from the existence of accessible cell line models as well as the characterization of genes affected by somatic mutations associated with specific human leukemias [3].

We have used a pair of factor-dependent murine cell lines to identify novel genes expressed within distinct hematopoietic lineages as an approach to the identification of novel candidate genes for development of diagnostic and therapeutic approaches to leukemia. The EML and MPRO cell lines were both established by infecting murine bone marrow cells with a retrovirus expressing a dominant negative retinoic acid receptor α (RARα) protein [4,5]. The infected cells were selected in the presence of either stem cell factor (SCF) or granulocyte/macrophage colony stimulating factor (GM-CSF). EML are SCF-dependent and resemble uncommitted hematopoietic progenitor cells. They can be induced to differentiate to the promyelocyte stage of granulopoiesis in the presence of interleukin-3 (IL-3) and high doses of all trans retinoic acid (atRA) [4,6]. Terminal neutrophil differentiation of EML cells can be induced by replacement of IL-3 and SCF with GM-CSF. MPRO cells are GM-CSF-dependent and can be induced to differentiate to neutrophils by adding high doses of atRA to the culture medium. The expression patterns of a number of genes expressed during hematopoiesis have been examined in EML and MPRO cells and generally agree with the patterns observed in other cell systems and in primary hematopoietic cells. Thus, EML and MPRO provide a powerful system for the identification and characterization of novel genes expressed within the hematopoietic lineage.

We have employed the representation difference analysis technique [7] to identify cDNAs representing genes expressed at higher levels in EML cells 72 hours after induction of differentiation than in uninduced cells. We describe the identification of a clone derived from an uncharacterized putative secreted protein. We have performed a comparative genomics analysis and determined that this protein is the founding member of an extended family of highly related proteins. This family contains three members in mammalian species, one or two members in invertebrate or simple vertebrate species and five or six members in fish. We have determined that one family member is a secreted glycoprotein and describe the expression pattern of the human and mouse genes in tissues and during hematopoietic differentiation.

## Results

### Identification of differentially expressed genes by representational difference analysis (RDA)

Total RNA was prepared from EML cells grown in the presence of SCF alone (0 hour) or in medium supplemented with IL-3 and atRA for 72 hours. The RNA was converted to cDNA and subjected to three rounds of RDA as previously described [6]. Six differentially-expressed clones were identified [6,8]. Clone number 1623 was chosen for further analysis and the differential expression of this gene was confirmed by Northern blot analysis. 1623 mRNA was essentially undetectable in the 0 hour sample but readily detectable in the 72 hour sample (data not shown and see figure 10).

### Sequence analysis of clone 1623

**Figure 1:**
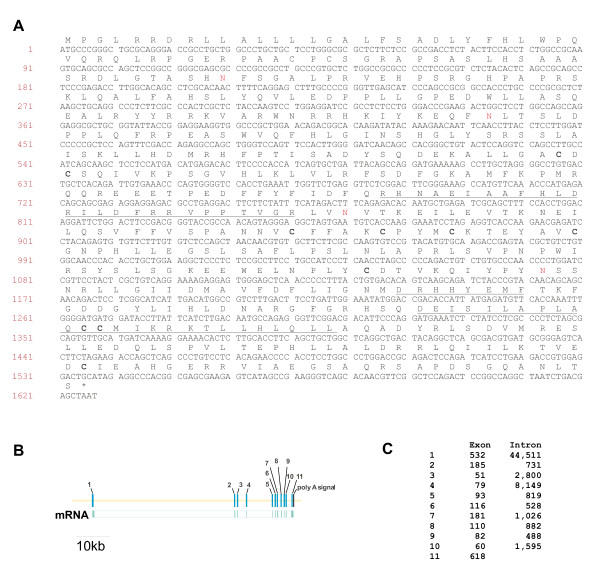
**Characterization of the full length mouse 1623 (Fam20a) cDNA and genomic sequence. **A. The full length cDNA derived from the original RDA clone was isolated using a combination of 5' and 3' rapid amplification of cDNA ends (RACE) procedures, comparisons to public databases, and amplification of putative full length clones by PCR. The full open reading frame was 1623 bp in length and encoded a 541 amino acid protein. The locations of regions conserved within the subsequently identified FAM20 family are indicated using underlines. Eight cysteine residues that are also conserved within the family are indicated in bold and four putative N-glycosylation sites are indicated in red type. B. The distribution of the 11 exons of the mouse Fam20a gene is shown with the exons indicated using numbers. A consensus polyadenylation signal is located downstream of the terminal exon. C. The sizes of the 11 exons and 10 introns of the Fam20a gene are shown.

The initial cDNA isolated by RDA was a 273 bp fragment that appeared to contain the coding sequence of the C-terminus of a protein that was not present at that time in public databases. To identify the full open reading frame of this cDNA, we first performed rapid amplification of cDNA ends (RACE) in both the 5' and 3' directions. Extension in the 3' direction revealed the presence of a consensus polyadenylation signal located 154 nucleotides downstream of the putative translation stop codon. Extension in the 5' direction yielded an additional 443 bp of sequence containing a contiguous ORF. Comparison of this extended sequence to public databases identified a cDNA (NM_017565) that was identical to clone 1623 in the region of overlap. This cDNA was isolated from a mouse mammary tumor but no functional analysis had been performed. We designed PCR primers based on the published sequence and confirmed that the cDNA isolated from EML cells was identical to the published sequence. The full length ORF was 1623 bp in length and encoded a protein of 541 amino acids (Figure 1A). The protein did not contain any recognizable motifs when examined using domain mapping software such as SMART or Profilescan (see Methods). However, a putative amino terminal signal sequence was identified using the SignalP analysis program. The cDNA mapped to an 11 exon gene located on mouse chromosome 11E1 (Figure 1B). The gene spanned approximately 60,000 bp of genomic sequence and the relatively large size of the gene is primarily due to the fact that the first intron is greater than 44,000 bp in length (Figure 1C).

### Identification of a family of related genes

The full length cDNA and the encoded protein were compared to sequences in public databases. We reported previously a weak similarity to a protein named Fjx1, which is the mouse orthologue of a Drosophila protein *named four-jointed *[8]. However, the degree of sequence identity between these proteins was low (16%) and thus the search was extended to include uncharacterized proteins. We first identified two other mouse proteins that displayed significant similarity to, but were distinct from, the query sequence. One protein (Accession number NP_663388 aka Riken C530043G21) was 409 amino acids in length and displayed 27% identity to the query sequence while the second (NP_085042) was a truncated version of a 579 amino acid protein that displayed 40% identity to the query sequence. We have subsequently discovered that these proteins are members of a highly related family and this family has received the official name "family with sequence similarity 20" (FAM20) from the Human Genome Organization Gene Nomenclature Committee. The protein derived from our original 1623 cDNA is named Fam20a in mouse and the other two family members are named Fam20b and Fam20c, respectively. Continued searches of public databases revealed the existence of related proteins in several other species. Each mammalian genome contains genes encoding three members that are orthologous to the three mouse proteins mentioned above. The accession numbers for the relevant cDNAs in human and rat are listed in Table 1 and we have also identified the same number of related sequences in other mammalian genomes, including the pig, cow and dog (data not shown). However, most of these sequences are incomplete and will not be described in detail here.

**Table 1:** Accession numbers for vertebrate FAM20 family members^1^

Family Member	Human	Mouse	Rat	Fugu	Zebrafish	Ciona
FAM20A	NM_017565	NM_153782	XM_221067 (BK001521)	SINFRUP00000146987 (BK001515)	ND	ND
FAM20B	NM_014864	NM_145413	XM_222770	SINFRUP00000138548 (BK001520)	CAI11712	AK115425
FAM20C	NM_020223	NM_030565	XM_221975 (BK001522)	SINFRUP00000140879 (BK001516) SINFRUP00000141732 (BK001518) SINFRUP00000163431 (BK001517) BK001519	ENSDARP00000009272 ENSDARP00000005688 ENSDARP00000028589	ND

1. Accession numbers refer to nucleotide sequences in GenBank except in the Fugu and Zebrafish listing where the accession numbers refer either to predicted peptides in the Ensembl database (entries beginning SINFRUP or ENSDARP) or Genbank (entry beginning CAI).

Entries beginning with BK refer to predicted sequences in the Third Party Annotation database arising from this study. ND: None detected.

**Figure 2:**
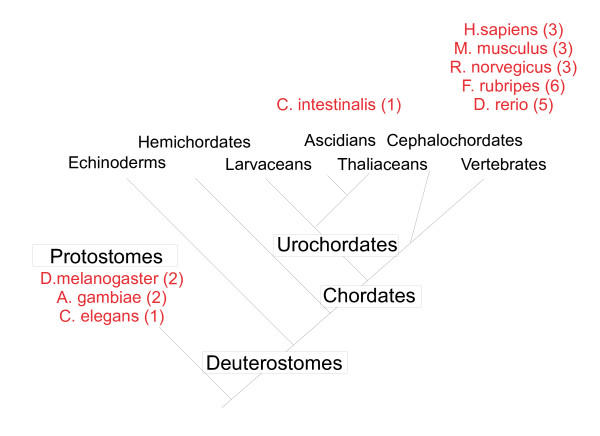
**Evolutionary distribution of FAM20 gene number. **An idealized evolutionary tree (modified from [10]) is shown with the number of FAM20 genes identified in several genomes as described in the text. The gene numbers are supportive of a single gene duplication event occurring in invertebrates (at least in insects) and multiple gene duplication events occurring in higher vertebrates.

In order to gain further information concerning the origin of the FAM20 family, we searched for related sequences in several other invertebrate and vertebrate organisms. The ascidian *Ciona intestinalis *is a model for a basal chordate organism and has emerged as a powerful model for evolutionary and developmental studies [9,10]. In particular, many gene families or subfamilies are represented by single members in *C. intestinalis *and thus the identification of an orthologue in this organism can provide useful information for evaluating the evolutionary origin of the members of a gene family. Consistent with this concept, we identified a single cDNA and the corresponding genomic locus in *C. intestinalis *that displayed significant sequence similarity to the mammalian FAM20 genes and proteins (Table 1). Complete genome sequences are also available for several invertebrate species and two related sequences were identified in *Drosophila melanogaster *and *Anopheles gambiae *with one family member in *Caenorhabditis elegans *(Table 2). Finally, analysis of genomic cDNA and protein databases for the pufferfish (*Fugu rubripes*) and zebrafish (*Danio rerio*) revealed the presence of six and five family representatives, respectively (Table 1). The gene numbers in these various species are listed on an idealized evolutionary tree in Figure 2 and suggest that the FAM20 gene family has undergone a complex set of gene duplications in both the invertebrate and chordate lineages.

**Table 2:** Accession numbers for invertebrate FAM20 family members^1^

Family Member	Drosophila	Mosquito	C. elegans
FAM20A	ND	ND	ND
FAM20B	NM_170079 NM_206490	EAA08010 EAA13434	NM_078126
Fam20C	ND	ND	ND

1. Accession numbers refer to nucleotide sequences in GenBank. ND: None detected.

### Assignment of subfamily relationships

**Figure 3:**
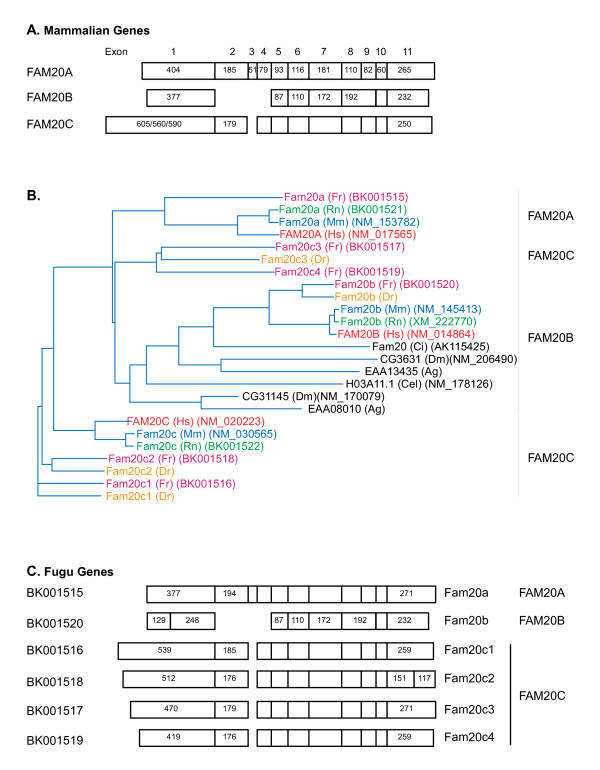
**Assignment of FAM20 family members to subfamilies. **A. Exon size and distribution of mammalian FAM20 members. The exons within each FAM20 gene in human, mouse and rat are indicated with the number of base pairs indicated within each exon. The sizes of exons that differ in size from the FAM20A genes are indicated. B. A dendrogram showing the relationships between FAM20 proteins from human (Hs), mouse (Mm), rat (Rn), *Fugu rubripes *(Fr), *Danio rerio *(Dr), *D. melanogaster *(Dm), *A. gambiae *(Ag), *C. intestinalis *(Ci) and *C. elegans*. The accession numbers of the cDNA sequences from which each protein sequence was derived are shown in parentheses except in the case of the mosquito family members where the accession number is used as the gene/protein name. Accession numbers for zebrafish peptide sequences are listed in Table 1. The FAM20 nomenclature has not been extended to the invertebrate sequences and the previous gene names have been used for Drosophila and C. elegans family members. The subfamily assignment of each family member is shown on the right. C. Exon number and size distribution within Fugu Fam20 members. The accession number of each sequence within the Third Party Annotation database is shown at left and family assignment based on dendrogram position and exon distribution is shown on the right.

To elucidate the nature of these putative gene duplications, we sought to assign the individual sequences from the various species into subfamilies based on protein sequence and gene structure, specifically using the number and size of exons in the latter case. Initially, the exon distribution of each of the Fam20 members in three mammalian species (human, mouse and rat) was compared and revealed obvious inter and intra orthologue similarities (figure 3A). Each FAM20A gene contained 11 exons and exon sizes were identical in these three species. Likewise, each FAM20B gene contained 7 exons that were identical in size in human, mouse and rat. The FAM20C genes each contained 10 exons and only exon 1 displayed any variation in size amongst these three species. In intra-orthologue comparisons, the exons in FAM20B and FAM20C genes clearly aligned with exons in the FAM20A genes, with small variations (in multiples of three bases) in the size of the internal exons in FAM20B. FAM20B lacks exons corresponding to exons 2–4 of FAM20A while FAM20C lacks exon 3. In addition, exons 8 and 9 in FAM20A and FAM20C are represented by a single exon in FAM20B that is identical in size to the combined exons in the other two genes. Thus, the three mammalian genes are highly evolutionarily related and presumably are derived from a common ancestral gene.

**Figure 4:**
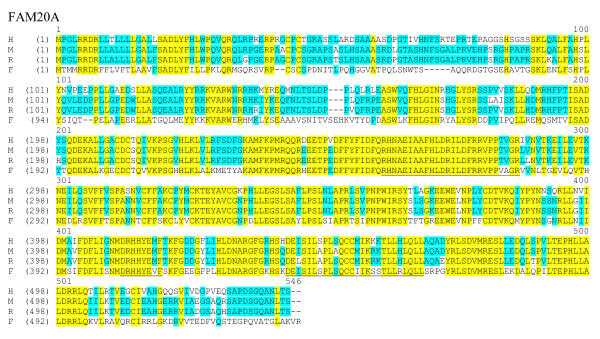
**Sequence alignment of FAM20A protein sequences. **The complete protein sequences of FAM20A members were compared using the AlignX component of the VectorNTI sequence analysis suite of programs. Identical amino acids are outlined in yellow, and similar residues are indicates in light blue. Conserved regions 1, 2 and 3 are underlined (see below). Gaps are indicated with dashes and the sequences are from human (H), mouse (M), rat (R) and puff erfish (F).

**Figure 5:**
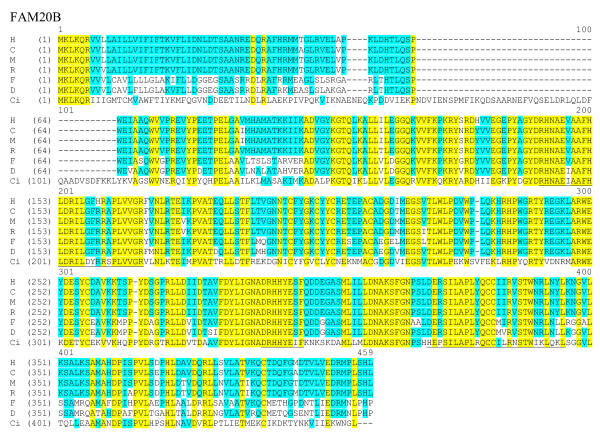
**Sequence alignment of FAM20B protein sequences. **The complete protein sequences of FAM20B members are presented as described in figure 4. The sequences are from human (H), mouse (M), rat (R), pufferfish (F), zebrafish (D) and *C. intestinalis *(Ci).

**Figure 6:**
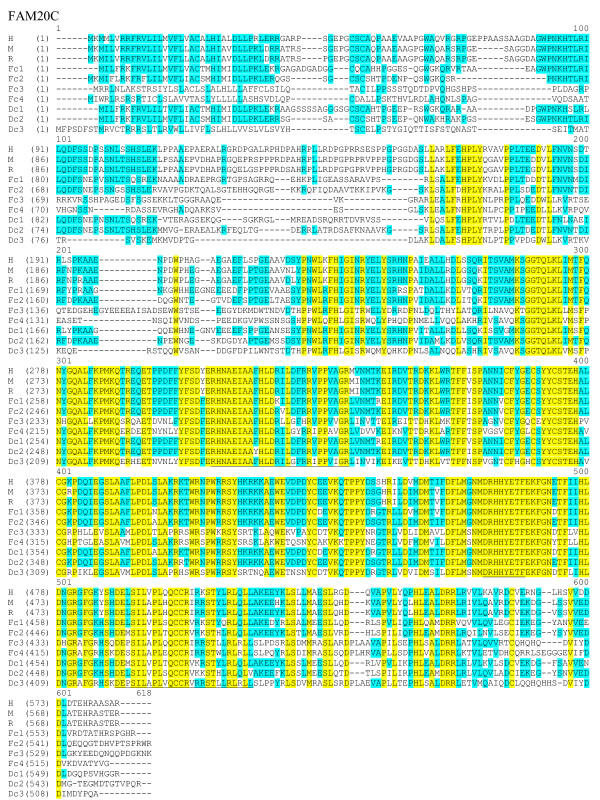
**Sequence alignment of FAM20C protein sequences. **The complete protein sequences of FAM20C members are presented as described in figure 4. The sequences are from human (H), mouse (M), rat (R), pufferfish (Fcl-4) and zebrafish (Dcl-3).

To assign the genes identified in other species to these three subfamilies, we performed a global comparison of the peptide sequences derived from 25 of the identified family members listed in Tables 1 and 2. One zebrafish protein (from FAM20A) was omitted as its sequence is incomplete. A dendrogram showing the results of this comparison is presented in Figure 3B. As expected, the mammalian orthologues clustered together and thus defined the subfamilies. All of the invertebrate proteins and the single protein identified in *C. intestinalis *clustered with FAM20B proteins, suggesting that this represents the ancestral branch of the FAM20 family. A single protein from Fugu and zebrafish clustered with the FAM20A and FAM20B family members while two Fugu and two zebrafish proteins clustered with FAM20C members. However, two Fugu proteins and one zebrafish protein clustered on a separate branch between FAM20A and FAM20B. In order to determine the subfamily to which these proteins belonged, we made use of the high degree of conservation of exon size and number noted in the mammalian genes (Figure 3C). The exon number and size of the Fugu and zebrafish genes encoding the two proteins assigned to FAM20A and FAM20B were consistent with their membership in these families. The only variations noted were a slightly larger exon 2 in the Fugu Fam20a gene and the division of exon 1 into two exons in the Fugu Fam20b gene. As in the mammalian family members, the sizes of the terminal exons varied more than the internal exons. The other four Fugu genes displayed exon distributions consistent with membership in FAM20C, despite the clustering of two of the encoded proteins between FAM20A and FAM20B. We have assigned each of these proteins to FAM20C with number suffixes (c1, c2, etc.) to designate individual genes and proteins. Each of these genes maps to distinct genomic loci and thus represents independent genes and not splicing variants of a smaller number of genes (data not shown). The gene structures of the three zebrafish family members were also consistent with this family assignment (data not shown). Comparisons of the derived protein sequences within each subfamily are shown in figures 4,5,6.

### Features of FAM20 proteins

**Figure 7:**
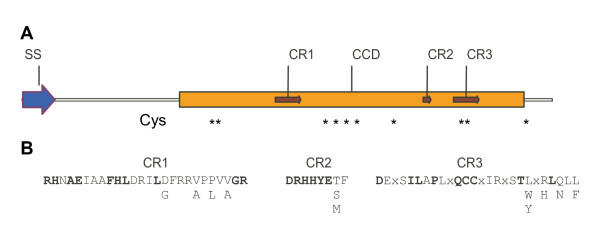
**Schematic representation of the structural features of FAM20 family members. **A. Structural features of FAM20A showing domains and residues conserved within the entire family. Key: SS: signal sequence; CCD: conserved C-terminal domain; CR: conserved region; Cys: cysteine residues conserved within CCD (indicated with asterisk). B. Consensus sequences were derived for CR1, CR2 and CR3 using a global comparison of all the family members listed in Tables 1 and 2. Residues that are invariant or only differ in one sequence are indicated in bold. Non-conserved residues are indicated with an x and positions with more than one common residue are shown below the main sequence.

All of the identified FAM20 protein sequences contain putative signal sequences at their amino termini but no other functional domains were unambiguously detected using several different annotation search software programs. In order to search for potential functional domains, we compared the sequences of all family members. These comparisons revealed that the greatest similarity was located within the carboxy-terminal two thirds of each protein (Figure 7A). We have named this region the conserved C-terminal domain (CCD) and it overlaps with a domain listed in the CDD database at NCBI as DUF1193. The CCD contains three distinct regions that are more highly conserved within all members of the family than the surrounding sequences (named conserved regions 1, 2 and 3 in figure 7A) and the consensus sequences for each conserved region were derived (figure 7B). Amino acids that are essentially invariant in all family members have been indicated in bold type and the heptapeptide DRHHYE in CR2 is the longest contiguous sequence that is conserved in all members of the family. A set of eight cysteine residues is also perfectly conserved within the CCD of each family member that may participate in inter-or intramolecular disulphide bond formation.

### Fam20a is a secreted protein

**Figure 8:**
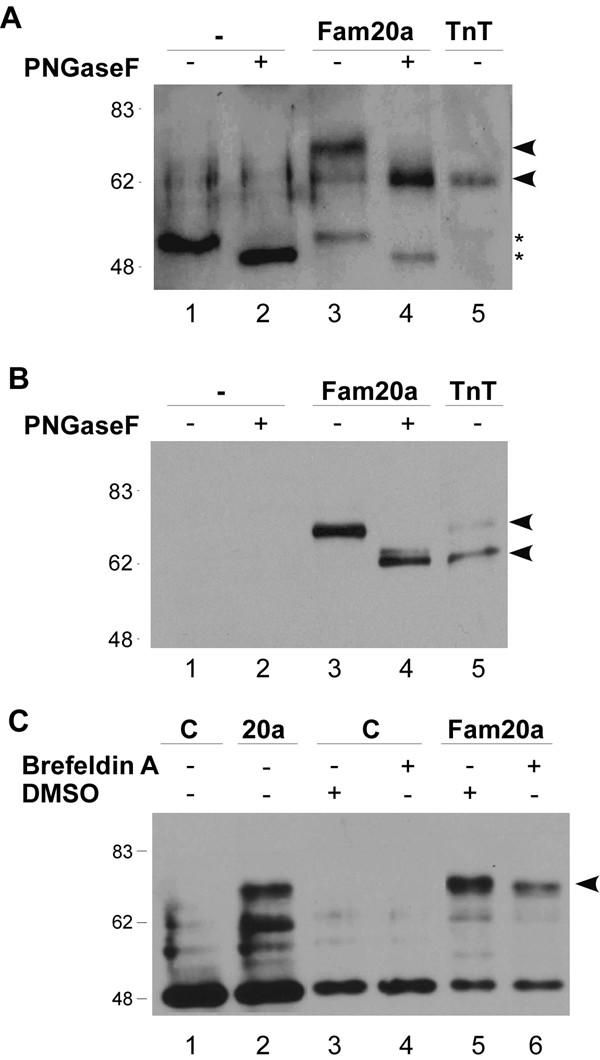
**Fam20a is a secreted protein. **COS-1 cells were transfected with either an empty expression vector (-) or one encoding mouse Fam20a with a C-terminal myc epitope tag and proteins were isolated from either the medium (panel A) or the cells (panel B). The proteins were analyzed by immunoblotting using a Myc tag-specific antiserum. Samples in lanes 2 and 4 of each blot were pre-treated with protein N-glycosidase prior to analysis to remove glycosyl groups. A recombinant form of Fam20a synthesized in rabbit reticulocyte lysates (TnT) was included on each gel as a size marker. The position of glycosylated and deglycosylated Fam20a is indicated using arrowheads and cross reacting material detected in the medium is indicated using asterisks. The location of molecular size markers is shown on the left of each gel. C. Protein samples from the medium of transfected cells that were untreated or treated with Brefeldin A were analyzed by immunoblotting using the Myc tag-specific antiserum. As Brefeldin A was resuspended in DMSO, the untreated cells were exposed to DMSO alone as a vehicle control. The amount of Fam20a detected in the medium of Brefeldin A treated cells was consistently lower than that observed in untreated cells (indicated using an arrowhead).

As the putative signal sequence was the only known domain identified in all family members, we next tested whether this sequence is functional. Signal sequences are commonly found on proteins that are directed to the endoplasmic reticulum (ER) and either retained there or processed and transported into the Golgi apparatus and secreted from the cell. Many proteins are glycosylated during their transit through the ER and Golgi apparatus and the mouse Fam20a protein contains four potential sites for N-glycosylation (indicated in red type in figure 1). As Fam20a does not contain an ER retention signal, we predicted that it should be detected in the medium of expressing cells. A mammalian expression vector was constructed that contained the full length mouse Fam20a coding sequence fused to a C-terminal Myc epitope tag and a hexahistidine sequence to permit purification. The plasmid was transfected into monkey kidney COS-1 cells and total protein was isolated from both the cells and the cell medium. Proteins in the cell medium were first processed on a Nickel column to isolate and concentrate the recombinant Fam20a protein and both protein samples were analyzed by immunoblotting using an antiserum specific for the Myc epitope. The predicted molecular weights of the full length and processed forms of Fam20a are 61,500 and 57,500, respectively, and a recombinant form of the protein synthesized in rabbit reticulocyte lysates was run alongside as a molecular size marker. The recombinant protein migrated just below the 62,000 mol.wt. size marker (Figure 8A and 8B, lane 5); however, the proteins detected in both the cell medium and cell extract migrated slower (lane 3). To test whether this slower migrating band represented a glycosylated form of Fam20a, the protein samples were treated with the enzyme N-glycosidase F (PNGaseF). The protein detected after enzyme treatment migrated more rapidly than the untreated protein and comigrated with the recombinant form of the protein (compare lanes 3, 4 and 5). We noted a second band that migrated slightly more slowly than the recombinant protein in the PNGase F treated cell extracts that may represent an alternatively modified form of Fam20a (Figure 8B, lane 4). To confirm that Fam20a is a secreted protein, we also exposed Fam20a-expressing cells to Brefeldin A, a fungal metabolite that specifically blocks transport from the ER to the Golgi apparatus, and examined the effects on Fam20a secretion. Brefeldin A treatment resulted in a consistent decrease in the amount of Fam20a detected in the cell medium (Figure 8C, compare lanes 5 and 6). Thus, Fam20a is a secreted glycoprotein.

### Fam20a secretion requires a functional signal sequence

**Figure 9:**
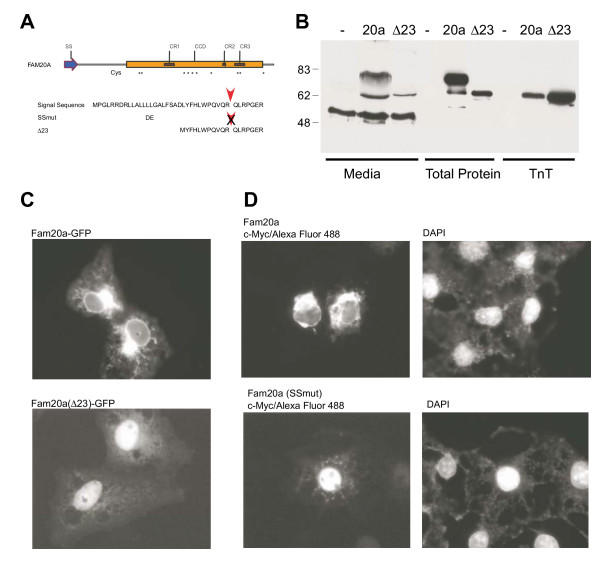
**Secretion of Fam20a requires an intact signal sequence. **A. Schematic representation of the putative signal sequence of Fam20a. The predicted cleavage site is indicated with a red arrowhead. The two amino acid substitutions introduced in the SSmut construct and the sequence remaining in the Δ23 mutant construct are shown. B. Immunoblot analysis of Fam20a and Fam20a(Δ23) protein levels in transfected COS-1 cells. The position of the glycosylated form of Fam20a (which is absent in Fam20a(Δ23) transfected cells is indicated with an arrowhead. C. Fluorescence images of COS-1 cells expressing either Fam20a-GFP or Fam20a(Δ23)-GFP. The wild type protein was observed within the cytoplasm, predominantly in a structure that is likely to be the ER. The mutant protein was primarily localized to the nucleus. D. Immunofluorescence images of Fam20a and Fam20a (SSmut) proteins as detected by antiserum directed against the C-terminal Myc epitope. The wild type protein was again detected in the ER and the mutant protein primarily in the nucleus. The cells have been counterstained with DAPI to delineate the nucleus.

We next tested whether the integrity of the signal sequence was required for Fam20a secretion. Signal sequences typically contain a high proportion of hydrophobic amino acids and 19 of the first 34 amino acids of Fam20a are hydrophobic (Figure 9A). Therefore, we expressed a Fam20a protein lacking the first 23 amino acids (FAM20a(Δ23)) in COS-1 cells and examined secreted and intracellular proteins by immunoblotting (Figure 9B). Glycosylated FAM20a(Δ23) protein was not detected in the medium (compare lanes 2 and 3) and immunoreactivity that comigrated with the unglycosylated recombinant protein was detected in the cell extract (lane 6). We also compared the subcellular location of the FAM20a(Δ23) protein to the wild type protein using GFP fusion proteins (figure 9C). The wild type Fam20a-GFP proteins displayed perinuclear and cytoplasmic staining consistent with ER localization. In contrast, the Fam20a (Δ23)-GFP protein was absent from the cytoplasm and appeared to be exclusively localized within the nucleus. To ensure that this effect was not a consequence of a gross change in protein structure due to the deletion of 23 amino acids, we also constructed an expression vector encoding a Fam20a protein with a two amino acid substitution within the putative signal sequence (Figure 9A). These changes (Leu^14^–Leu^15 ^to Asp-Glu) were predicted to disrupt the signal sequence without grossly altering the protein structure. Again the mutant protein displayed nuclear staining and was absent from the ER (Figure 9D). These results confirm that an intact signal sequence was necessary for secretion of Fam20a and that secretion was accompanied by prominent localization of the protein to the ER.

**Figure 10:**
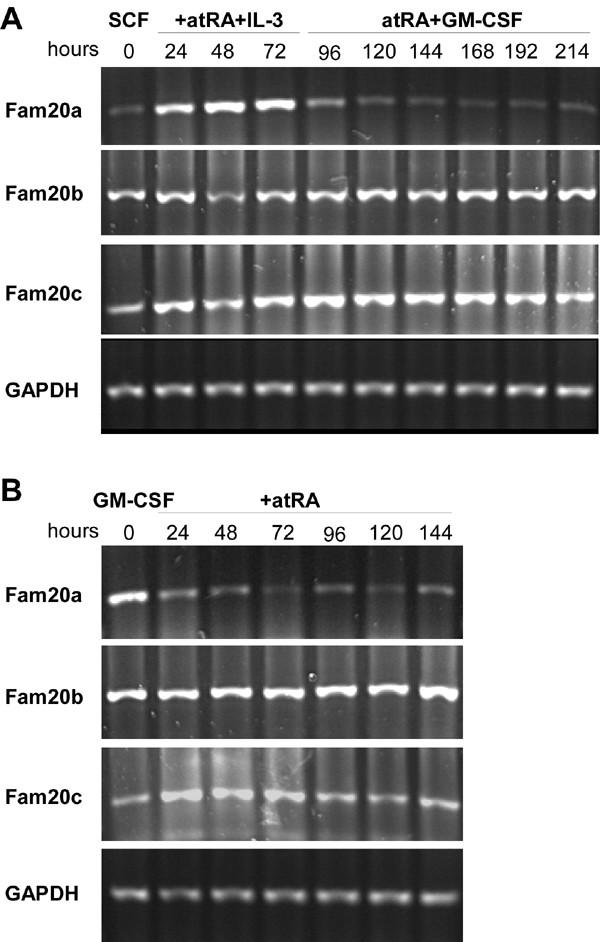
**RT-PCR analysis of mouse Fam20 mRNA levels during differentiation of EML and MPRO cells. **Total RNAs were prepared from EML (panel A) or MPRO (panel B) cells at the indicated timepoints during myeloid and granulocytic differentiation. cDNAs prepared from each sample were amplified using primer pairs specific to each mouse family member. The PCR products were analyzed by agarose gel electrophoresis and stained using Gelstar SYBR Green DNA stain. GAPDH was used as a loading control.

### Expression patterns of FAM20 genes during myeloid differentiation

We originally identified Fam20a as a differentially expressed mRNA in EML cells induced to differentiate along the myeloid lineage. To determine whether Fam20b and Fam20c are also expressed during hematopoiesis, we performed RT-PCR analysis of cDNAs prepared at various times during experimentally-induced differentiation of EML and MPRO cells using primers specific to each family member. Fam20a mRNA levels were low in uninduced EML cells maintained in the presence of SCF and increased during the subsequent 72 hours of incubation in atRA and IL-3 (Figure 10A). EML cells mature to the promyelocyte stage of neutrophil differentiation under these conditions and can subsequently be differentiated into neutrophils by adding GM-CSF in place of SCF and IL-3. Fam20a mRNA levels decreased during terminal neutrophil differentiation in EML cells and also in MPRO cells induced to undergo the same differentiation process in the presence of atRA (Figure 10A and 10B). Fam20b and Fam20c mRNAs were readily detected in both cell lines and their levels did not vary dramatically during the differentiation process in either cell line (Figure 10A and 10B).

### Expression patterns Of FAM20 genes in human tissues

**Figure 11:**
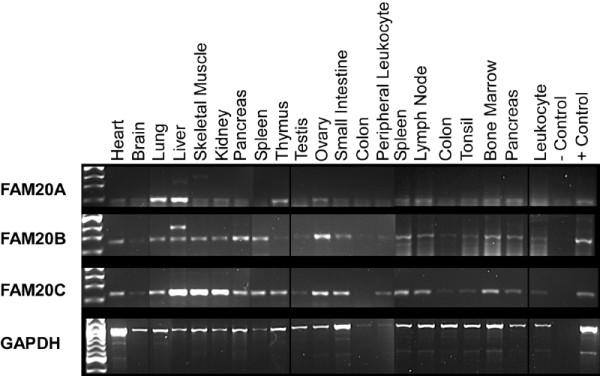
**RT-PCR analysis of human FAM20 mRNA levels in human tissues. **A panel of commercially available human cDNAs prepared from the indicated tissues was analyzed by PCR using primer pairs specific for each of the human FAM20 family members. GAPDH was again used as a loading control although large variations were observed in the GAPDH signal in the different tissues.

Although we originally isolated Fam20a from a hematopoietic cell line, cDNAs and ESTs derived from each of the FAM20 family members have been isolated from non-hematopoietic tissues (data not shown). Therefore, we examined the expression patterns of the three genes in a panel of cDNAs derived from various human tissues. FAM20A displayed the most restricted expression pattern with high levels in lung and liver and intermediate levels in thymus and ovary (Figure 11). Low levels of FAM20A mRNA were detected in several other tissues. FAM20B and FAM20C were expressed in a wider variety of tissues and their expression patterns were very similar.

## Discussion

Several classes of secreted proteins, including the colony stimulating factors or hematopoietins, are important regulators of hematopoietic differentiation and function [11]. These molecules are of clinical significance due to their use in stimulating hematopoiesis in patients with neutropenias and other hematological disorders [11]. Consequently, the identification of novel secreted proteins that display specific spatiotemporal expression patterns in hematopoietic cells is of great interest. In this report, we describe the identification and initial characterization of a new family of secreted glycoproteins expressed within the hematopoietic lineage as well as several other cell types and tissues. The family has been named FAM20 to indicate the fact that the members are related by sequence similarity rather than a specific shared function and contains three members in mammals. We anticipate that the members will acquire new names as their specific functions are determined. The family contains three separate subfamilies which are referred to as FAM20A, FAM20B and FAM20C in humans.

### FAM20 proteins: features and potential functions

At the present time, the specific function(s) of the FAM20 proteins is unknown. Analysis of the sequences of the proteins from various species failed to reveal obvious similarities to known functional domains except for an N-terminal signal sequence. Expression studies clearly demonstrated that the mouse Fam20a protein is a secreted protein and that disruption of the signal sequence prevented the detection of the glycosylated form of the protein in cell media. Surprisingly, disruption of the signal sequence resulted in redistribution of intracellular Fam20a from a cytoplasmic compartment that is likely to be the ER to the nucleus. It is unclear whether this redistribution is functionally significant and is presumably due to the presence of a cryptic nuclear localization signal (NLS) in the protein. NLSs are generally comprised of stretches of basic residues [12] and several candidate regions rich in basic amino acids are present in Fam20a that could direct the mislocalized protein to the nucleus.

Overall, the FAM20 proteins vary in length between 400–670 amino acids with the FAM20B family members being the shortest and the FAM20C members generally being the longest. The variation in length is due to differences in the length of the less conserved N-terminal region. The FAM20 proteins are further characterized by a highly conserved C-terminal region of approximately 350 amino acids that we named the conserved C-terminal domain (CCD). We noted the presence of three regions that are more highly conserved amongst all family members, with the most invariant extended sequence being the DRHHYE heptapeptide within conserved region 2. The function of the peptide is as yet unknown but three possible functions can be proposed. First, the histidines within this sequence could be involved in the coordination of metal ions that may be required for FAM20 protein function. Second, FAM20 proteins may be enzymes and this sequence may be a component of the active site of the enzyme. Interestingly, a weak match was detected between this region of certain FAM20 family members and a conserved domain within the phosphatidylinositol 3- and 4-kinases [13]; however additional experiments must be performed to address the relevance of this similarity. Third, the highly charged nature of this peptide suggests that it may be located on the surface of FAM20 proteins, where it may participate in protein:protein interactions. Although we have been unable to identify any other proteins in public databases that contain the exact sequence, the sequence RHHYE is found between amino acids 41–45 in the N-terminal region of the viral infectivity protein (Vif) from human immunodeficiency virus 1 (Accession number AAQ09611) [14]. Vif enhances HIV-1 infectivity by blocking the antiviral activity of the nucleotide editing enzyme APOBEC3G [15]. Vif exerts its inhibitory effect by binding to and inducing the degradation of APOGEC3G and also by blocking its translation [16,17]. The APOBEC3G interacting domain is located within the N-terminal region that contains the FAM20-related pentapeptide although the specific involvement of this sequence has not yet been investigated [16]. Thus, this sequence within conserved region 2 may be a site for protein:protein interaction. However, APOBEC3G or related proteins are unlikely candidate binding partners as they are intracellular proteins. We also noted that one of the Drosophila FAM20 proteins (NM_170079; CG31145) was identified as a protein that interacted with the Dynein light chain protein Dlc90f [18]. Dyneins are motor proteins involved in intracellular transport [19]. It appears unusual that a secreted protein would directly interact with a motor protein; therefore, this may represent a specific interaction of this particular family member in fruitfly.

### Evolution of the FAM20 gene family

Tremendous progress has been made over the past decade in the sequencing of genomes from species at different positions on the evolutionary tree [20-25]. This vast amount of information can be used for comparative studies to elucidate the evolutionary origin of members of gene families. We have reported here the identification of orthologues of the FAM20 members in two insect species (*D. melanogaster and A. gambiae*), a simple chordate (*C. intestinalis*), three mammals (*H. sapiens, M. musculus and R. norvegicus*) and two fish (*F. rubripes and D. rerio*). In each case, the identification of these genes as *bona fide *transcription units is supported either by direct experimental evidence for the human and mouse genes, or by the existence of multiple EST sequences in public databases. A single FAM20 gene was identified in *C. intestinalis*, which is considered to be a representative of a basal chordate related to the common ancestor of humans and other higher chordates [10]. The *C. intestinalis *genome encodes approximately 16,000 genes and generally contains single representatives of superfamilies in higher vertebrates, thereby permitting the elucidation of likely evolutionary origins of genes within these families [9]. The C. intestinalis FAM20 protein clustered with the FAM20B subfamily members, as did the family members identified in invertebrate species. Therefore, we propose that the FAM20B subfamily contains the direct descendents of the ancestral FAM20 gene and that the FAM20A and FAM20C subfamilies result from duplication and subsequent evolution of this ancestral gene [26]. This pattern is consistent with the 2R hypothesis of genome evolution proposed by Ohno in 1970 [27]. In the FAM20 case, the loss of one gene at some stage of higher vertebrate evolution would need to be hypothesized to explain the final paralogue number of three in mammals. A single round of gene duplication appears to have occurred subsequent to the divergence of the nematode and insect lineages, giving rise to the two paralogues in fruit fly and mosquito that both cluster within the FAM20B subfamily.

A further round of gene duplication has been proposed to have occurred in fish [28] and the existence of five to six FAM20 genes in pufferfish and zebrafish is consistent with this hypothesis. However, it is interesting that the expansion appears to have occurred exclusively in the FAM20C subfamily. This pattern could be explained either by two successive rounds of gene duplication of a small genomic region that included the original FAM20C representative, or by two rounds of duplication of larger genomic segments followed by gene conversion of FAM20A and FAM20B descendents (or parents) to yield four FAM20C members. Analysis of the genomic regions surrounding each of the FAM20C genes in fish will be necessary to distinguish between these two possibilities. Nevertheless, the expansion of the FAM20C subfamily in fish suggests that these proteins have acquired specific functions required within these species.

### FAM20 expression patterns in mammalian cells and tissues

Expression analysis of the three FAM20 family members in mammalian tissues and hematopoietic cells showed that FAM20A is expressed in a much more restricted pattern that the other two members. Importantly, Fam20a was also the only member to display obvious differential expression in hematopoietic cells undergoing myeloid differentiation. Fam20a mRNA levels were highest during intermediate stages of differentiation of EML cells, at a time when many cells are becoming committed to the myeloid lineages and undergoing extensive proliferation [4,8,29]. EML cell cultures also give rise to a small number of cells from B cell and erythroid lineages under these conditions but these cells do not survive after SCF and IL-3 is removed from the medium and replaced with GM-CSF. Thus, the decrease in Fam20a mRNA levels after the 72 hour timepoint could be interpreted in two ways. First, Fam20a may be expressed in one of the lineages that cannot survive in GM-CSF. Second, Fam20a may be expressed specifically in cells committed to the myeloid lineage and its expression may decrease during terminal granulocytic differentiation. The similarities in Fam20a expression patterns in EML cells after the 72 hour timepoint and in MPRO cells suggests that the second explanation is most likely. Therefore, we propose that Fam20a is primarily expressed in cells committed to the granulocytic lineage and presumably plays a role in either lineage commitment of cell proliferation. The hypothesis is currently under investigation using gene disruption techniques and through further characterization of the Fam20a protein.

## Methods

### Cell culture and Representational Difference Analysis (RDA)

EML and MPRO cells were cultured as described previously [6,30]. Briefly, EML cells were cultured in Iscove's modified Dulbecco's medium containing 20% horse serum (Atlanta Biochemical, Norcross, GA) supplemented with 10% BHK-MKL conditioned medium (CM) as a source of stem cell factor (SCF). The cells were differentiated by adding 10% of WEHI-3 CM as a source of IL-3 and all trans retinoic acid (atRA, 1 × 10^-5 ^M, Sigma) for 72 hours. Terminal granulocytic differentiation was induced by removing IL-3 and SCF and adding 10% BHK-HM5 CM as a source of granulocyte/macrophage-colony stimulating factor (GM-CSF). MPRO cells were cultured in Dulbecco's modified Eagle's medium supplemented with 10% fetal bovine serum (FBS, Hyclone, Logan, UT) and 10% BHK-HM5 CM. Terminal differentiation was induced by adding 1 – 10^-5 ^M atRA to the culture medium. For collection of RNA samples at different timepoints, 10 cm dishes were seeded with 150,000 cells and cultured under identical conditions. Cells were harvested from individual wells at 24 hour intervals for RNA preparation.

Total RNA was prepared from isolated cells using a modified guanidium isothiocyanite/phenol extraction procedure as described previously [31]. Poly A^+ ^RNA prepared from EML cells at zero hours (stem cell stage) and 72 hours (promyelocyte stage) was converted to double stranded cDNA and three rounds of RDA was performed as described previously [6]. cDNA clones representing six putative differentially expressed genes were identified and named according to clone number. Initial characterization of these clones was described earlier [6,8] and clone number 1623 was examined further in this study.

Cos-1 cells were maintained in Dulbecco's modified Eagle's medium (DMEM, BioWhittaker, Walkersville, MD) containing 10% FBS and Penicillin/Streptomycin (BioWhittaker) under standard cell culture conditions. For transfections, cells were plated at a density of 2.5 × 10^4 ^per well in six well plates and transfected using the Effectene Transfection Reagent (Qiagen, Valencia, CA) under conditions recommended by the manufacturer.

### Isolation of a full length 1623 cDNA

Analysis of the sequence of the original 1623 cDNA isolated from the RDA experiments revealed that it contained the coding sequence from the C-terminus of an uncharacterized protein. 5' and 3' rapid amplification of cDNA ends (RACE) was performed using Marathon-Ready cDNA from mouse spleen using the Advantage cDNA PCR kit (both from Clontech) as described previously [30]. The RACE reactions were performed using gene specific primers designed based on the original 1623 sequence or on sequences identified in early RACE reactions and adaptor primers provided with the cDNA. The 5' RACE products were compared to sequences in public databases and identified a mouse cDNA (NM_017565; aka DKFZp434F2322) that was identical to the extended 1623 sequence. PCR primers were designed that overlapped the putative initiation and stop codons of the ORF encoded by NM_017565 and used to PCR amplify products from cDNAs prepared from undifferentiated MPRO cells. The product of this PCR reaction was subcloned and sequenced in its entirety. The sequence was identical to the original clone (which was isolated from mammary tissue) indicating that the same mRNA is expressed in both tissues. The full length clone (now named Fam20a) encoded a 541 amino acid protein.

### Sequence analysis

All basic sequence manipulations were performed using the VectorNTI suite of sequence analysis programs (Informax/Invitrogen Corp., Carlsbad, CA). Identification of FAM20 members from different species was initially achieved using standard BLAST searches at NCBI . Genome specific searches were performed either through NCBI or Ensembl  or through genome specific websites (*Ciona intestinalis*: ; *Fugu rubripes*: ). Searches were performed either as nucleotide-nucleotide (blastn) searches or as protein-translated nucleotide (tblastn) searches. In general, these searches were sufficient to identify gene regions encoding the most highly conserved regions of the proteins, particularly in the fish and Ciona genomes. To assemble complete genes, genomic regions containing the identified regions of similarity were downloaded into VectorNTI and searches were performed for individual exons, initially derived from mammalian genes but subsequently, from potential orthologues in fish or lower vertebrates, depending on the sequence being examined. Putative exons were examined for the presence of consensus splice donor and acceptor sites and complete sets of exons were assembled into cDNAs for translation and further comparisons. These results were compared against genes assembled by two gene prediction programs, FGENESH:  and GENSCAN: , however, these programs often made incorrect assignments that required manual assessment of the results. The protein sequences derived from each of the examined species were primarily used in the comparisons to define subfamily assignments and alignments were performed in the AlignX program in VectorNTI using the blosum62 scoring matrix. Protein domain searches were performed using the Simple Modular Architecture Research Tool (SMART)  and/or Profilescan . Signal sequence searches were performed using the SignalP analysis program [32]. Sequences derived by annotation of genome sequences in this study have been submitted to the Third Party Annotation database at NCBI under accession numbers BK001515 to BK001522. The FAM20 family name has been approved by the Human Genome Organization nomenclature committee.

### Plasmid construction

The Fam20a and Fam20aΔ23 were constructed in the pEGFP-N1 vector by PCR cloning. *Xho*I and *Hind*III restriction sites were engineered into the PCR primers (Fam20a: 5'GGCCTCGAGGCCATGCCCGGGCTGCGCAGG3', 5'GGCAAGCTTGCTCGTCAGATTAGCCTG3'; Fam20aΔ23: 5'GGCCTCGAGGCCATGTACTTCCACCTCTGGCCG3' and reverse primer was as above). Similar strategy was used to clone Fam20a and Fam20aΔ23 in the pcDNA3.1 vector (Forward primers were same as above; Fam20a and Fam20aΔ23 reverse primers: 5'GGCAAGCTTGGGCTCGTCAGATTAGCCTG3'). The Fam20a(SSmut) was generated using a PCR-based site-directed mutagenesis kit (Quickchange, Stratagene). All of the sequences were verified by sequencing using an ABI automated sequencer.

### Western blotting

Whole cell extracts were prepared from transfected COS-1 cells by lysing directly in 2X Laemmli Sample Buffer as described previously [31]. Purified His-tagged proteins were prepared as described below and mixed with 2X Laemmli Sample Buffer (120 mM Tris-HCl (pH 6.8), 10% Glycerol, 3.3% SDS, 0.2 M dithiothreitol, 0.004% Bromophenol Blue). Equal amounts of total cellular and purified secreted proteins were separated by 12% SDS-PAGE and transferred to nitrocellulose membranes (Micron Separations, Westborough, MA). Membranes were blocked in 5% non-fat milk in TBST (100 mM Tris-HCl (pH 7.5), 0.9% NaCl, 0.1% Tween-20) for 1 hour. Myc-tagged Fam20a proteins were detected using an anti-myc mouse monoclonal antibody (Santa Cruz Biotechnology, Santa Cruz, CA). Horseradish peroxidase-conjugated donkey anti-mouse antibody (Promega, Madison, WI) was used for the secondary antibody. The immune complexes were detected using SuperSignal chemiluminescence detection kit (Pierce, Rockford, IL). In vitro transcribed and translated Fam20a was generated using the TnT T7 coupled reticulocyte lysate system (Promega) as described previously [33].

### Purification of His-tagged proteins from cell media

COS-1 cells were transfected as described above and media were collected after 48 hours and mixed with freshly made 10X binding buffer (500 mM NaH_2_PO_4_-H_2_O (pH 8.0), 150 mM NaCl, 10 mM Imidazole). His-tagged proteins were purified using Ni-NTA Magnetic Agarose Beads (Qiagen, Valencia, CA) according to manufacturer's recommendations. Following the purification step they were either used directly in western blot analysis or for deglycosylation experiments. Purified proteins were subjected to enzymatic deglycosylation using the GlycoPro deglycosylation kit (ProZyme, San Leandro, CA) following the manufacturer's protocol.

### Immunofluorescence

COS-1 cells were plated on glass coverslips and transfected with the indicated constructs the following day. After 48 hours of incubation, the cells were fixed in 1% formaldehyde for 30 minutes at room temperature. Coverslips were incubated in PBS (137 mM NaCl, 2.7 mM KCl, 4.3 mM Na_2_HPO_4 _(pH 7.3)) containing 1% Triton X-100 for 10 minutes to permeabilize the cell membranes. They were then transferred to PBST (PBS containing 0.1% Tween-20) and incubated for 30 minutes. Mouse monoclonal anti-Myc antiserum (Santa Cruz) was used at 1:2,000 dilution in PBST containing 1% bovine serum albumin (1 hour at room temperature). Coverslips were then washed with PBST three times and incubated with the secondary antibody (Alexa Fluor 488 [Molecular Probes, Eugene, OR]) for 1 hour. Coverslips were washed three times in PBST and once in water for 15 minutes each, and were mounted on glass slides. The cells were observed by epifluorescence microscopy using an Axiovert 135 TV microscope (Zeiss, Gottingen, Germany). Images were captured with a Kodak DC290 zoom camera and analyzed with MetaMorph 6.0 (Universal Imaging Corporation, Downingtown, PA).

### Reverse transcription-polymerase chain reaction

Total RNA was purified as described above from EML and MPRO cells at 24 hour timepoints during the differentiation process of each cell line The primers were designed using the Vector NTI sequence analysis suite of programs (InforMax, Frederick, MD) and were follows: MmFam20a forward 5'-catagaggcccacggcgagcg-3' and reverse 5'-atggaatggggcaacag gggc-3'; Mmfam20b forward 5'-tggacaggattctgggtttc-3' and reverse 5'-ccagggatgtcgatgtttct-3'; MmFam20c forward 5'-agcagacgagagagcaggag-3' and reverse 5'-cggatctccttggtcatgtt-3'; HsFAM20a forward 5'-ctggcaggaaaagagtg-3' and 5'-cccgaacttggtgaacatct-3', HsFAM20b forward 5'-ccctgaagagacaccagaagagc-3' and reverse 5'-gaaacccagaatcctgtcca-3', HsFAM20c forward 5'-ggctcacgttccacattggt-3' and reverse 5'-aaagtcagggggtgtctcct-3'.; mouse glyceraldehyde-3-phosphate dehydrogenase (GAPDH) forward 5'-aatggtgaaggtcggtg tgaac-3' and reverse 5'-gaagatggtgatgggcttcc-3'; human (GAPDH) forward 5'-tgaaggtcggagtcaacggatttggt-3' and reverse 5'-catgtgggccatgaggtccaccac-3'. GAPDH was used as control for cDNA integrity. Single stranded cDNA was reverse transcribed from 2 μg of total RNA using 400 Units of Superscript RNase H^- ^Reverse Transcriptase (Invitrogen, Carlsbad, CA), 0.125 mM dNTPs, 10 mM DTT, and 1 μM oligo (dT)_15 _in a total volume of 50 μl for 1 hour at 37°C. 2 ul of the RT reaction was then mixed with 1 ul of 10X PCR Buffer (500 mM Tris-HCl (pH8.3), 2.5 mg/ml crystalline BSA and MgCl_2 _at 10, 20 or 30 mM (Idaho Technology Inc., Idaho Falls, ID), 0.2 mM each dNTP, 0.05 μM of each primer and 1.25 Units Taq DNA polymerase (Fisher Scientific, Pittsburgh, PA) in a total volume of 10 μl. Reactions were loaded into a capillary tube and PCR cycles were carried out using the Rapidcycler Thermal cycler (Idaho Technology). Annealing temperatures and Mg^2+ ^concentrations were initially optimized for each primer. A commercial set of tissue cDNAs, (Human Multiple Tissue cDNA (MTC) Panels, Clontech, Palo Alto, CA) was used for the RT-PCR analysis of human FAM20 family members.

## List of Abbreviations

FAM20: family with sequence similarity 20; EML: Erythroid, myeloid, lymphoid cell line; MPRO: mouse promyelocyte cell line; GM-CSF: granulocyte/macrophage-colony stimulating factor; PHSC: pluripotent hematopoietic stem cell; HGF: hematopoietic growth factor; RAR: retinoic acid receptor; atRA: all trans retinoic acid; SCF: stem cell factor; IL-3: interleukin-3; RDA: representational difference analysis; RACE: rapid amplification of cDNA ends; CCD: conserved C-terminal domain; GFP: green fluorescent protein; CM: conditioned medium.

## Author Contributions

DN isolated full length Fam20a cDNA, performed transfection experiments and drafted the manuscript. HY performed RT-PCR assays. IN assisted in construction of expression vectors. SS performed genomics analyses. EC and EGB provided reagents and assistance in performing RT-PCR assays. YD performed the original representational difference analysis and SCW directed the project and performed genomics analyses. SCW also wrote the final draft of the manuscript and all authors read and approved this version.
